# Pet Ownership in Aotearoa New Zealand: A National Survey of Cat and Dog Owner Practices

**DOI:** 10.3390/ani13040631

**Published:** 2023-02-11

**Authors:** Rachel Forrest, Leena Awawdeh, Maria Pearson, Natalie Waran

**Affiliations:** 1 Te Pūkenga—Eastern Institute of Technology, Hawke’s Bay, 501 Gloucester Street, Taradale, Napier 4112, New Zealand; 2 School of Veterinary Science, The University of Queensland, Gatton, QLD 4343, Australia

**Keywords:** Aotearoa, cats, companion animal, dog, knowledge, New Zealand, practice, pet owners, survey

## Abstract

**Simple Summary:**

Currently, there are very few studies examining pet ownership in Aotearoa, New Zealand (NZ) and the factors that influence pet owners’ practices. To facilitate better pet management and enhance companion animal welfare, it is vital to understand current pet owners’ practices and the factors that influence pet owners’ behaviors. This knowledge will inform effective human behavior modification interventions that will benefit animal welfare and ensure pets in NZ are living a good life. This study investigates pet owners’ practices in their households, using an online survey conducted between January and March 2019 involving adults residing in NZ. In addition, we explore what veterinary care pet owners access. There was a total of 2744 participants in the survey, with 2385 respondents answering the pet ownership questions. Of these, 885 (37%) owned both cat/s and dog/s, while 652 (28%) and 609 (26%) owned cat/s only or dog/s only, respectively. Data analysis using the demographics of the respondents provides insights into some of the factors associated with differences in the pet owner practices highlighted by the survey.

**Abstract:**

This study used an online survey distributed between January and March 2019 to adults residing in Aotearoa New Zealand (NZ) to investigate cat and dog owner practices. Of the 2385 respondents, 885 (37%) owned both cat/s and dog/s, while 652 (28%) and 609 (26%) owned cat/s only or dog/s only, respectively. Nine percent of respondents (n = 212) did not own a cat or dog when the survey was administered. Gaps were identified in the practices of NZ pet owners with regard to regular grooming, immunizations, and deworming treatments. It was also found that many pets, especially cats, were allowed to wander freely both inside and outside the house. Collectively, these gaps in practice raise parasitology and infection concerns which may impact negatively on animal welfare and may increase the prevalence of zoonotic diseases. This study also revealed the need to improve desexing practices, particularly in dogs. Respondents in the survey expressed the wish to have pets regardless of the financial strain they may impose, indicating that future research should focus on reducing the financial burden of pet ownership along with promoting positive pet ownership practices. Our findings suggest the need for better education resources about pet ownership which are easily accessible and target diverse populations. The findings of this study will aid in developing appropriate educational resources to promote animal welfare and increase pet-related knowledge among the NZ populace.

## 1. Introduction

Companion animal pet ownership in Aotearoa, New Zealand (NZ) has been estimated to be 64% of households, according to Companion Animals New Zealand (CANZ) [1]. This ranks NZ second, following the United States of America (USA), with 67% of the households owning companion animal pets [2]. Currently, cat/s (41% of households) and dog/s (34%) are the most frequently owned pets in NZ. Approximately three-quarters of NZ cat (74%) and dog (78%) owners regard their pets to be family members [1].

Globally, pet–human interactions have been the center of many research studies due to the complexity of the bond [3,4,5,6,7,8]. Many studies have demonstrated that having a pet improves physical and emotional health and the well-being of the owner [9,10,11,12]. For example, some studies reported that having pets decreases anxiety depression and increases the owner’s physical activity levels [10,13]. On the other hand, some research explores the negative impact of human–animal interaction on mental and physical health and well-being that can occur due to owning pets, such as the risk and fear of contracting a zoonotic disease, the risk and fear of physical injuries (e.g., bites, scratches) and increased financial burden [4,6,11,14,15,16,17]. Yet, Marino and Lilienfeld suggest that having pets does not affect the owner’s health outcomes [18]. Furthermore, several factors with regard to the owner (e.g., owner gender, age, socio-economic status, knowledge, and attitudes, etc.), pet (e.g., breed, age, individual characteristics of the pet, etc.) and surrounding environment in which the person and pet reside affect the complex bond between humans and their pets [19,20].

Regardless of the health and well-being impacts for the pet’s owner, the owner should be encouraged to ensure their pet are able to “express a rich behavioral repertoire, use their abilities, and fulfil their potential through active engagement with their environment” [21]. This definition can be expanded further using a one welfare lens, whereby a pet living a good life should not be detrimental to the welfare and wellbeing of other animals, humans, or the natural environment. The fact that pets are an integrated part of the community means their presence impacts their owner and other people, and this has led to increasing concern about pet ownership [10,22]. Issues regarding pet ownership in this wider context emerge mainly due to a lack of owner knowledge regarding their pet’s needs and behaviors [23]. Several international studies have found that attitudes towards pet ownership were associated with several factors, such as gender and education level [14,24,25,26,27]. Irresponsible pet ownership has been associated with increased free-roaming and stray animals, and pet overpopulation [24,28,29]. Even though there are guidelines and policies that people should follow as pet owners in NZ, there needs to be more research investigating how these extend beyond the responsibilities toward the animal itself and into broader society and within the one welfare context. In keeping with this, it is important to identify owner and community knowledge gaps regarding the accountable and responsible management of pets.

Currently, there are very few studies examining pet ownership in NZ and the factors and variables that influence pet owners’ practices considering the wider range of identities and beliefs in NZ, which may influence these activities [30]. To facilitate better pet management and companion animal welfare in NZ, we must not just comprehend why people act as they do towards their pets, but we also need to explore methods for enhancing the understanding of our communities about improving pet ownership practices. The current study interrogates the relevant data collected from the Furry Whānau Wellbeing project funded by the New Zealand Companion Animal Trust (NZCAT) [31] to explore NZ pet owners’ practices to maintain the health and wellbeing of their pets, and what demographic factors influence these behaviors. Our study’s findings will contribute to the evidence base that will help inform interventions aimed to improve knowledge about pet ownership. This, in turn, will facilitate positive outcomes for national animal welfare, and human health and wellbeing while also taking environmental impacts into consideration.

## 2. Materials and Methods

The data was collected as part of the 2019 NZCAT *Furry Whānau Wellbeing* research project [31] with ethical research approval obtained from the Research and Ethics Approval Committee of the Eastern Institute of Technology (REAC ref. 19/53). The overarching intent of the survey was to gather information to help inform companion animal welfare initiatives within a one welfare context (Supplementary Figure S1). Data was collected via an online survey that was developed in consultation with local Māori representatives and internationally recognized topic experts. The online survey was administered in English via Survey Monkey^©^ from 8 January 2019 to 31 March 2019 and was open to residents of NZ 18 years of age or older. The survey collected demographic data including: gender, ethnicity, age group, region as defined for local government purposes, residence type (urban, rural, etc.), childhood residence (urban, rural, etc.), household income range, highest qualification (education level), and household make-up (number of children and number of adults). A detailed description of the survey development, questions, and data collection method is provided in Ref. [31]. The questions in the survey related to demographics can be found in Supplementary Table S1. Below are the questions related to the practice of cat and dog owners:

- Do you own a dog? Yes, No.
- How many dogs do you own? 1, 2, 3, 4, Other (please specify).
- Is your dog allowed inside the house? Yes, No.
- If yes, when are they allowed inside? Tick all that apply: Whenever they choose (free access) during the day; Whenever they choose (free access) at night; My dog is always confined indoors during the day; My dog is always confined indoors at night; Only when someone is at home; Other (please specify).
- What do you allow your dog/s to do inside? Tick all that apply: Roam freely; Sit or sleep on the furniture; Sleep on or in your bed; Remain in a designated area, e.g., crate, pet bed, or kennel; Eat food from the bench/table; Sit on the bench/table; Other (please specify).
- When your dog/s are outside, they are (tick all that apply): Chained up; Free-roaming and can leave the property; In a dog run/pen; In a kennel; Free-roaming on a well-fenced property; Other (please specify).
- Which of these apply to your dog/s? Tick all that apply: My dog vaccinations are up to date; My dog is desexed; My dog is flea treated according to product instructions; My dog is wormed according to product instructions; My dog’s nails are clipped when needed; My dog’s teeth are cleaned by the vet when needed; My dog’s teeth are cleaned by me when needed; My dog is groomed (brushed or clipped) regularly; My dog is groomed (brushed or clipped) occasionally; My dog has caused me financial stress (e.g., vet bills); My dog is a pedigree; Please provide further explanation if required.
- Do you own a cat? Yes, No.
- How many cats do you own? 1, 2, 3, 4, Other (please specify).
- Is your cat allowed inside the house? Yes, No.
- If yes, when are they allowed inside? Tick all that apply. Whenever they choose (free access) during the day; Whenever they choose (free access) at night; My cat is always confined indoors during the day; My cat is always confined indoors at night; Only when someone is at home; Other (please specify).
- What do you allow your cat/s to do inside? Tick all that apply: Sit or sleep on the furniture; Sleep on or in your bed; Eat food from the bench/table; Sit on the bench/table; Remain in a designated area (room, crate); Other (please specify).
- Which of these apply to your cat/s? Tick all that apply: My cat vaccinations are up to date; My cat is desexed; My cat is flea treated according to product instructions; My cat is wormed according to product instructions; My cat’s nails are clipped when needed; My cat’s teeth are cleaned by the vet when needed; My cat’s teeth are cleaned by me when needed; My cat is groomed (brushed or clipped) regularly; My cat is groomed (brushed or clipped) occasionally; My cat has caused me financial stress (e.g., vet bills); My cat is a pedigree; Please provide further explanation if required.

### Data Analysis

To analyze the qualitative data, the inductive thematic analysis was undertaken independently by at least two researchers, and then the results were compared and consolidated to provide the final themes [32]. Both descriptive and inferential statistics (IBM SPSS Statistics version 25) were used to explore the quantitative data, and these are described in detail in previous publications [31,33,34], along with a full demographic description of the survey respondents and an investigation of the associations between various demographic factors and variables using z-tests, correlations and general linear mixed effects models [31]. For this particular study, survey responses were described using percentages, and forward stepwise binary regressions were used to explore what demographic factors may be associated with a positive response for a particular pet care statement. An association was indicated by a statistically significant (*p* < 0.05) odds ratio (OR) with an OR > 1 indicating the factor or variables was associated with increased likelihood of a positive response and an OR < 1 indicating association with a decreased likelihood of the factor or variable being associated with a positive response.

## 3. Results

### 3.1. Demographic Description of the Respondents

As mentioned, a detailed demographic description of all the respondents (n = 2744), along with the demographics of those that are cat and those that are dog owners, can be found in our previous publications [31,33,34]. Among the survey respondents, there was an under-representation of males (7.7% of the respondents; national representation in the NZ 2018 Census was 49.4%) and Māori (8.3% of the respondents; national representation in the NZ 2018 Census was 16.5%). Many of the local government regions had no or very few Māori and/or male respondents, and for this reason, region was not included in any of the inferential statistical analyses.

### 3.2. Cat and Dog Owner Practices

Only some respondents answered every question, as they were able to skip those questions they did not wish to answer or that were not relevant to them. Not all respondents owned a pet at the time of the survey, though they had done so previously. There were 2358 respondents that answered the questions on pet ownership practices. Of these, 885 (37%) owned both cat/s and dog/s, while 652 (28%) and 609 (26%) owned cat/s only or dog/s only, respectively. When completing the survey, 212 respondents (9%) did not own a cat and/or dog.

#### 3.2.1. House Access and Confinement Practices

Almost all respondents indicated they would allow their dog (96%, n = 1449/1512) and or cat (99%, n = 1524/1534) inside their house; however, the extent of access varied (Table 1). Some of the respondents chose to leave a comment to clarify their choice of responses, and several themes emerged from these comments (Supplementary Tables S2 and S3). Cats and dogs were often allowed free access inside during the day when someone was home. Dogs were either outside, crated inside, or had restricted access (e.g., laundry or garage) when not supervised. The latter was also true for dogs at night. For cats, unsupervised access depended on whether or not there was a cat door. If a cat door was not available, cats were often left in or out during the day, depending on where they were when the owner departed. For some cats left inside there was restricted access to certain areas of the house (e.g., the conservatory or garage). Another emergent theme was that cats and dogs were often kept inside during inclement weather, illness or fireworks. Other cat owners opted to keep their cat/s indoors at all times to keep their feline companion safe and to protect prey animals such as birds and lizards, while some owners noted that their cats would not come inside at all. Two respondents differentiated between farm/working dogs and pet dogs, with farm dogs being kept exclusively outside while the pets’ dogs were kept indoors.

The majority of dog owners indicated that their dog/s could roam freely when inside (88%, n = 1278/1449) and that their dog/s were free to use the family furniture (65%, n = 945/1449) and sleep on or in their owners’ bed (56%, n = 813/1449, respectively). Only 19% (n = 278/1449) of respondents restricted their dog/s to designated areas while inside. A minority of respondents allowed their dog/s to eat food from and sit on kitchen surfaces (1.2%, n = 18/1449 and 0.8%, n = 12/1449, respectively). Many of the respondents chose to leave a comment to clarify their responses. The major themes were that most dogs had their own furniture (e.g., couches and beds), most dogs required an invite or permission to use human furniture and that smaller dogs enjoyed more inside access via pet doors and more inside freedoms than larger dogs. This is evidenced by the following representative quotes, “They have beds for sleeping, but they are allowed on our bed if we tell them they can get up for fuss and attention”, “The big dogs are only allowed up when we call them up” and “Big dog not allowed on furniture/bed”.

Most respondents (89%, n = 1335/1449) indicated that when their dog/s are outside they are free to roam on a well-fenced property. Of these, 12% (n = 157/1335) also selected kennel, run and or chained up and where a comment was provided indicated that their dogs were only free to roam the property when someone was home. For example, “Run with kennel when alone, free-roaming in fenced boundary when we’re home”. Five per cent (n = 75/1449) of respondents indicated that their dog/s were allowed to roam free and could leave the property at least some of the time. Of these, 27 commented that their dog/s “don’t leave the property”, with some stating it was because the dogs “know they are not allowed” and “will get growled at if they wander off the property”. Several of the respondents indicated they were from a rural property and that although the dog/s could leave, they didn’t; for example, “we live very rural, [my dog] doesn’t leave the property” and “They [my dogs] roam free on our farm, but do not leave the property”.

Nearly all respondents indicated that when their cat/s were inside, they were free to use the family furniture (97%, n = 1488/1527) and sleep on or in their owner’s bed (91%, n = 1388). Only 3% (n = 42/1527) of respondents restricted their cat/s to designated areas while inside. A minority of respondents allowed their cat/s to eat food from and sit on kitchen surfaces (12%, n = 186/1527 and 22%, n = 334/1527, respectively). Again, many of the respondents chose to leave a comment to clarify their responses. The major themes were that most cats could “Go wherever they please except on surfaces where food is prepared or eaten” but that it was “Difficult to tell/train as they [cats] tend to do as they please, when they please—haha” and “Technically they are not allowed on the bench…but cats really don’t give a damn about rules”. The presence of a dog was typically indicated as a reason as to why a respondent’s cat was allowed on the bench/table. For example, “The cat eats from the bench because of the dogs as they will eat the cat food” and “They have a designated spot on the bench so the dog can’t eat their food”. Some cats had designated rooms at night. For example, “Has his bed in own room”, “They are not allowed in the bedrooms at night. They have an outdoor cattery to give them safe outdoor space away from other cats and wildlife” and “Husband allergic so [the] bed’s banned”.

#### 3.2.2. Dog Care Practice

Table 2 summarizes the responses from 1499 owners about several aspects of dog care. The majority of respondents (>80%) indicated that their dog/s were vaccinated, wormed, flea treated, desexed and had their nails clipped. Grooming and teeth cleaning had lower positive response rates, but many of the respondents left comments indicating that their dog/s was short-haired and did not require grooming and or that their dog’s diet naturally cleaned their teeth. For example, “Doesn’t need grooming—short-haired—nails kept down by lots of walking and teeth by using Dentastix and bones”. There was a strong theme that any “Financial stress is worth it!”, “He is old and required surgery. It was expensive but worth it.” Several respondents indicated that they had pet insurance. Not surprisingly, as household income increased, the likelihood of financial stress due to pet ownership responsibilities decreased.

Gender was found to impact worming practices, with females being more likely (92%) to worm their dog/s according to the product instructions, compared to males (86%) and gender diverse (75%). As age of the respondents increased, there was a decreased likelihood of the dog/s being flea treated according to product instructions and the owner cleaning their dog’s teeth. Ethnicity also influenced the likelihood of professional teeth cleaning, with fewer Māori dog owners including this in their pet care practices when compared to NZ European and other ethnicities (36% vs. 45% and 44%, respectively). As household income increased, the owner’s likelihood of cleaning their dog’s teeth decreased, and the likelihood of professional teeth cleaning increased. Interestingly, as the qualification (education) level increased, the likelihood of professional teeth cleaning decreased.

Ethnicity also influenced desexing practices with fewer Māori owning a desexed dog compared to NZ European and other ethnicities (desexing 94% vs. 97% and 98%, respectively). Having a rural upbringing also decreased the likelihood of a dog being desexed. In contrast, if the owner currently lived in a town or city, there was an increased likelihood of the dog being desexed and regularly groomed. As the number of adults in the house increased, the dog was less likely to be desexed. Likewise, with an increasing number of children in the household, there was a decreasing likelihood that the dog/s were desexed.

#### 3.2.3. Cat Care Practice

Table 3 summarizes the responses from 1536 owners about several aspects of cat care. The majority of respondents (>90%) indicated that their cat/s were wormed, flea treated, and desexed. As with dogs, several of the respondents said they only carried out flea and worm treatment as required, for example, “Flead and wormed when required, not as a matter of course” and “Short-haired & indoors so don’t get fleas or worms”. More than three-quarters (77%) confirmed that their cat’s/cats’ vaccinations were up to date. There was a perception of kitten vaccinations being important but adult ones being less of a priority, with some of the respondents saying, “we do not do annual vacs but everyone has been vaccinated twice as kittens” and “My cats have all had initial vaccines”. Fewer than half of the respondents selected yes for the remaining statement concerning teeth cleaning, nail clipping, and grooming, with some of the respondents saying they had not been necessary or that their cat/s were not compliant, for example, “Won’t let me clip nails & I refuse to sedate them for this so carpet suffers!”, “She keeps her claws short on trees outside and doesn’t like being brushed do only gets it occasionally!” and “Haven’t required nail or teeth treatment”. Nineteen percent of respondents acknowledged that their cat had caused them financial stress, for example, “One of our cats has a breathing issue, she’s also been hit by cars a few times. Although money is a worry, she’s worth every cent”. Several respondents indicated they would put their cat before themselves, “I would go without so that they don’t have to”.

Gender influenced selection choices for ‘My cat is wormed according to product instructions’ (male 82% versus females 91%) and ‘My cat has caused me financial stress’ (males 9% versus females 19%), with males being less likely to select these. Ethnicity was not found to be associated with the choice selections for each of the statements. An increasing age range was associated with a decreasing likelihood that a cat/s were being flea treated as instructed by the product, having up-to-date vaccinations, being occasionally groomed, having their owner clean their teeth, and causing financial stress. Increasing household income was associated with an increased likelihood that the cat/s teeth were cleaned, and their nails were clipped by a veterinarian. Conversely, increasing household income was associated with a decreasing likelihood of causing financial stress and the owner cleaning their cat’s teeth. Qualification level influenced whether cat/s were desexed, and their teeth were cleaned by a veterinarian. Furthermore, if the cat was a pedigree, all of these increased in likelihood as qualification level increased. Those brought up rurally were less likely to select ‘My cat vaccinations are up to date’ (73% versus 78%), and ‘My cat’s teeth are cleaned by the veterinarian when needed’ (37% versus 47%) and more likely to select ‘My cat’s teeth are cleaned by me when needed’ (12% versus 8%). Those respondents currently living in a town or city were more likely to have cat/s that were flea treated according to the product instructions (92% versus 86%), had their teeth cleaned by a veterinarian when needed (46% versus 40%) and their nails clipped (37% versus 31%). As the number of children in a household increased, the likelihood of the cat/s in the household being desexed decreased, as did the likelihood the cat/s were wormed, had their teeth cleaned by a veterinarian, had their nails clipped, were regularly groomed and had caused financial stress. As the number of adults in a household increased, there was a decreased likelihood of a respondent’s cat/s causing financial stress.

The following comment summarized the collective sentiments of many with regard to veterinarian checks and flea and worm treatments,

*They should have veterinarian checks and flea and worm treatments etc. when needed. But if you have experience in what to look for health-wise, i.e., keeping an eye on their weight, energy, behaviour changes, water consumption, physical changes etc., and don’t want to use chemicals on them all the time (flea worm treatments etc.) I don’t think it should have to be a constant thing to do, as long as you’re checking and looking out for them and get them treatment/checked with any concerns or if it’s been a long period since they were checked or they are elderly etc.*

Several of the respondent’s comments highlighted the perception that worming and flea treatment may not always be necessary; for example, “In Southland, fleas are not a problem so we don’t use a flea treatment. We would if our dog/s needed one though” and “Worming should be given on the advice of a veterinarian to avoid resistance”. Vaccinating young dog/s was viewed as important and some thought adult’s dog/s were over-vaccinated; for example, “Puppy vaccination are super important but we over vax adult dog/s.” One respondent commented that “Veterinarian care where I live is all owned by one company. It’s very expensive and they often pressure people into unnecessary vaccinations and products”.

Another common theme in the comments was that all “…breeders should be registered, and all other dogs should be desexed to prevent the number of strays and puppy-farms around”. A common perception regarding desexing was that it was being performed when the animal was too young; for example, one respondent wrote, “Desexing is done far too early; again, the evidence shows you should wait for the dog/s to fully mature, so their hormones have settled and they’ve finished growing, usually around two years of age.” One respondent went further and wrote,

*I have read that it’s best that dogs are desexed after their growth plates have closed, around 18 months which is best for the dog/s if the owner is responsible. However, that’s not always the case, so it would be great to see ovary-sparing-spay and vasectomy rather than traditional ops on younger dog/s so they can keep their hormones needed for correct growth.*

## 4. Discussion

This study investigated NZ pet owners’ practices and the findings have identified several common practices that if changed could ensure pets are living a good life within the broader one welfare context. Our findings indicated that NZ pet owners value having pets and view them as being beneficial to the household despite the fact that approximately one-fourth of respondents reported that owning a cat and or dog creates financial strain. This supports earlier research demonstrating the presence of beneficial impacts of pet ownership on the owner’s quality of life [35,36,37]. However, several of the findings in this study raise concerns, which could have negative health (animal and human) and environmental (including conservation) implications.

The present study found that both cats and dogs were often allowed to roam freely in the house, a finding that is consistent with some overseas studies [38,39]. Interestingly, we also found that both canine and feline pets, especially cats, were allowed to sit and feed on the kitchen benchtops and sleep in their owner’s beds. A recent study by Thomas and Feng has highlighted the risk of pets and their owners contracting foodborne illnesses from incorrect storage, preparation and handling of pet food and the need for owner education about separation of human and animal food preparation and eating areas to mitigate the risk [37]. Likewise, co-sleeping with pets has also been associated with an increase the risk of human illness, along with increased sleep disturbance [40]. However, for the pet owner there are also positive psychological (bonding, comfort, fall asleep easier, feel protected) and physical benefits of bed sharing which reinforce this [40,41]. Unfortunately, these sort of practices can also enhance the emergence of zoonosis among the community [42,43]. Therefore, it is essential to educate pet owners about zoonotic diseases and their transmission to help to reduce the health risks impact to both companion’s pets and humans. Such education would be timely given the context of the COVID-19 pandemic, as some studies suggest that cat/s act as intermediate hosts and contribute to the indirect transmission of human virus SARS-CoV-2 while, on the other hand, dog/s do not [43,44].

One zoonoses of particular concern globally and to NZ is toxoplasmosis. This disease is caused by the protozoan parasite *Toxoplasma gondii*, which can infect all birds and mammals (including humans) as its intermediate hosts. Infection occurs via the consumption of raw meat or milk contaminated with *T. gondii* tissue cysts, or via the consumption of raw vegetables or water contaminated with *T. gondii* oocysts shed in cat feces [45]. *T. gondii’s* definitive host is the cat which was introduced to NZ in the 18th Century [46]. Infection in humans can have severe consequences if the person is immunocompromised or pregnant, leading to impaired eyesight in the immunosuppressed and miscarriage or the birth of children with mental retardation and blindness, respectively [45]. Toxoplasmosis also represents a significant cost to the livestock industry in NZ as it causes embryonic, fetal and neonatal death in both sheep and goats, thereby requiring the use of regular vaccinations to manage the disease. Furthermore, toxoplasmosis has been reported in several native NZ species, including shellfish [47,48], Hector and Māui dolphins (endangered) [46], the NZ sea lion (endangered) [49], and various bird species including the kākā and red-crowned kākāriki (both endangered), the Kererū and kiwi [46], some with fatal consequences. Thus, better management of pet cats along with stray and feral cats is imperative to reduce the health and conservation risks posed by *T. gondii*. The risk of toxoplasmosis alone presents a strong argument for all cats to be kept enclosed. Pets with outdoor access and contact with other animals have an increased risk of parasite infection and or infestation [43,50,51].

Recently, Pennelegion et al. conducted an online survey in the United Kingdom (UK) to investigate the deworming practice among dog/s and cat/s owners, although the authors did not explore the respondent demographics association with deworming practice [52]. The results of Pennelegion et al. suggested that UK pet owners do not follow the recommended treatment frequencies, which may result in inadequate external and internal parasite treatment, resulting in serious health issues for the pets, parasite resistance and increasing the zoonotic risk [52]. Similar owner attitudes toward parasite treatment and prevention protocols were documented in Netherlands, Germany and France [53,54]. Interestingly, the majority of respondents surveyed in this study indicated that their dog/s and/or cat/s had outside access and that they were wormed and treated for fleas in accordance with the product instructions. Regarding the latter, the respondents may have interpreted the question as relating to the application of the product and not the recommended frequency of use. In future studies, these aspects should be asked independently of each other. Nevertheless, in this study, increased pet owner age and being a male were associated with not following worming and flea treatment products instructions. This is consistent with research that suggests that females are reportedly more empathetic towards animals [55], are more interested in health-related topics, and are primarily responsible for the health care of their pets compared to males [56]. Given that inadequate and inconsistent deworming practices can have serious health and wellbeing implications for both pets and owners [53,57], it is important to better understand the gender differences in pet owner practices, so that appropriate interventions can be put in place. The association with increasing age is interesting and raises questions about the accessibility to pet care for older adults, and it is worthy of further investigation.

The parasitology concerns raised in the above discussion emphasizes the responsibility of veterinary clinics to effectively implement one welfare measures to control and prevent both zoonotic and non-zoonotic infestations and to ensure that pet owners are adequately educated on the importance of appropriate external and internal parasite treatments, frequency and administration methods according to evidence-based guidelines. The results of this survey indicate this may be an issue as a number of respondent comments highlight the lack of trust and pet owners’ perception that veterinarians are motivated by money rather than the best care for their pets [31]. This contradicts the 2020 Companion Animals NZ Report, which surveyed NZ pet owners and found that veterinarian clinics were considered the most reliable source of information [1]. The latter is in agreement with a 2016 Canadian research in which 216 pet owners participated in a focus group, and the conclusion was that pet owners rated their veterinarians as the most reliable source of information about pet health [58]. Nevertheless, there may be a need for non-veterinary clinic-based education in NZ about parasite treatment for cats and dogs, especially targeted at male pet owners and older people.

Interesting differences in the vaccination rates between dog/s and cat/s were uncovered in this study. Approximately three-quarters (76%) of cat and 91% of dog owners confirmed that their pets’ vaccinations were up to date, results which agree with previous studies [59,60]. However, higher cat vaccination rates (86%) were reported in Italy [61]. These results are perhaps not surprising given the incorrect owner perception that was reported in this study regarding the importance of vaccination for their cat health. This finding is alarming because, according to Companion Animals NZ’s last report [1], more than 84% of cat/s roam freely outside, exposing them to a number of feline infectious diseases agents such as feline Panleukopenia and feline respiratory viruses, and feline immunodeficiency virus [62,63,64]. Similar owner attitudes toward dog/s were documented in Germany, where the author found that puppies are usually up to date with their vaccinations [65]. The author suggested that the finding was a reflection of the owner’s lack of knowledge regarding the importance of establishing their pets’ immunity and maintaining this immunity [65]. Furthermore, infection of pet cats with non-zoonotic agents increases their risk of infection with zoonotic pathogens [66]. Collectively, the research indicates that as well as creating a significant animal welfare concern, unvaccinated pets are also a potential public health concern.

Overpopulation is another animal welfare and public health concern, along with being an environmental and conservation issue as predation level increase. Cat and dog overpopulation has been attributed to relinquishment and abandonment of pets, as well as to birth rates, with the latter emphasizing the importance of desexing [67]. Previous research has reported that life experience, cultural differences, and socioeconomic status influence the attitude of dog owners towards spaying and neutering [59,68]. In this study, almost all cats were desexed (97%), whereas only 87% of dogs were desexed. Identifying as Māori and or having a rural upbringing decreased the likelihood of a dog being desexed. Additionally, as the number of adults in the house increased, the dog was less likely to be desexed. In contrast, if the owner currently lived in a town or city, there was an increased likelihood of the dog being desexed. This finding differs from McKay et al., where the owner’s place of residency did not affect their pet’s neuter status [59]. However, the data collected in McKay et al. involved in-person interviews in the urban area of Auckland, and may not accurately represent residents of rural areas. The current study did not find a correlation between respondent gender or age and desexed status of their dogs, although other NZ and international studies have found such difference [59,69,70].

Recent studies have shown that the appropriate desexing age varies between different dog breeds and can range anywhere from six months up to two years, depending on the age at which dogs reach puberty [71,72,73]. Even though a number of educational campaigns are conducted throughout NZ to educate pet owners about the importance of desexing their animals as early as possible, our findings suggest the need for dog breed-specific and culturally appropriate education targeting specific cohorts of the NZ population. In addition, the regulation of pet adoption should be modelled after those of other nations. For instance, many states in the USA have strict desexing practices that requires all dogs to be spayed or neutered before they can be adopted out, even if this occurs before the age of six months [74].

In agreement with other studies, the majority of respondents (80.7%) got their dog’s nails clipped when needed [24,25,28,35]; however, only 35.5% of cat owners did this. Unfortunately, our survey did not distinguish between nail trimming performed by the owner or veterinarian, as it did for teeth cleaning. Regular nail trimming can limit the damage caused by the natural scratching behaviors of cats, mitigating some of the consequences of problem behaviors that can lead to cats being relinquished [75]. Interestingly those households with higher incomes and that were in an urban area were more likely to have their cats’ nails trimmed when needed. One possible theory is that this reflects situations where a cat is more likely to live indoors and the household items being scratched potentially being of higher quality or more expensive. However, it is more likely that these findings reflect that cat owners are not confident trimming their pet’s nails and that those with higher incomes and that live in urban areas have better access to veterinarians to do this for them. More research needs to be conducted to confirm these notions and investigate what motivates owners to have their cat’s nails trimmed regularly and what are the barriers, for example, poor conditioning to nail trimming procedures, and excess distress to cat and owner.

As with nail clipping, teeth cleaning, whether performed professionally or by the owner themselves, was also more likely occur for dogs (73.9%) than cats (53.0%). Not surprisingly, households with greater earnings were more likely to get their pets teeth professionally cleaned, unless from a rural upbringing, and then the owner was more likely to do it. The respondent age also influenced teeth cleaning practices of the owner cleaning their cat’s or dog’s teeth. For dogs, ethnicity also influenced the likelihood of professional teeth cleaning, with fewer Māori owners including this in their pet care practices compared to NZ European and other ethnicities (36% vs. 45% and 44%, respectively). Interestingly, as the qualification level increased, the likelihood of professional teeth cleaning of cat teeth increased but dog teeth decreased. This may suggest pet owners are more confident cleaning the teeth of dogs than cats (which are often less than compliant) making dental care cats potentially costlier as owners are reliant on veterinarians. This notion is supported by the observation that the household income was positively associated with professional teeth cleaning. These results align with a recent study finding that indicate that cat owners have relevant information about the dental health of their pets, but that preventative actions are not taken often enough to promote excellent oral health in cats [76]. Negative association has been reported between periodontal diseases and teeth cleaning [77,78], highlighting the need to increase NZ pet owners’ understanding and use of dental care. This is in agreement with UK data about the impact of inadequate owner education creating dental issues in older cats, which leads to welfare concerns. Our findings emphasize the significance of regular veterinary consultations for enhancing pet owner knowledge and boosting their confidence about their pet’s dental needs as well as implementing home education programs on cat and dog dental care.

The study data demonstrate approximately double the dog/s owners performing essential grooming for their dog/s (nails clipping and coat brushing) in comparison to cat/s owners. Less than half of the cat respondents reported they performed teeth cleaning, nail clipping, and hair grooming, with some of the respondents saying the grooming practice had not been necessary or that their cat/s were not compliant. These findings suggest that the primary welfare difficulties for cats in the NZ are related to a lack of information or awareness of cat welfare requirements and safe handing techniques. Consequently, cat behavioral problems may arise that result in owners relinquishing their pet, cat suffering, and or cat illness such as periodontal disease [76,77]. These finding suggests the need for further research to identify gap in the knowledge to develop an owner education resources, especially for cat owners targeting these particular welfare issues, for example, safe handling techniques for dental care and grooming. Furthermore, these education resources need to be accessible to lower-income and rural-dwelling cat owners, and therefore, veterinary clinics may not be well placed for delivery.

## 5. Strengths and Limitations of This Study

Although the online survey enabled more respondents from across NZ, which was one of the study’s strengths, the self-selected responders were disproportionately more female respondents, and Māori were underrepresented when compared to NZ demographics [79]. Thus, the survey sample was not representative of the general populace. The preponderance of female participants was anticipated, since this is typical of the internet survey method [80,81]. To complete the survey required literacy and digital technology skills, which also may have prevented those pet owners lacking these skill from participating if they did not have someone willing to assist them. In addition, pet management practices are likely to be determined at the household level as opposed to by individual thus the responses might not have reflected the prefer ownership practices of the respondent. Furthermore, the statistical analyses are exploratory and are indicative of where more research could be undertaken that is designed to answer specific questions about the impact of a certain factor or factors on a particular practice or practices. Consequently, our results should be interpreted in light of all these limitations.

## 6. Conclusions

New Zealanders value their pets regardless of the financial difficulty they may cause. Nevertheless, household income was found to influenced several pet ownership practices such as teeth and nail care but, interestingly, not vaccination or desexing. Future research should, therefore, examine methods to minimize the cost burden of pet ownership. Several gaps were identified in the practices of NZ pet owners’ with regard to regular grooming, immunizations, and deworming treatments. It was also found that many pets, especially cats, were allowed to wander freely both inside and outside the house. Together, these gaps in practice raise parasitology and infection concerns which may impact negatively on animal welfare (the pet’s welfare along with that of other domesticated animals and wildlife) and may also increase the risk of zoonoses becoming both a public health. Our findings highlight the need to improve accessibility to veterinary services and culturally appropriate educational resources, especially for lower-income households, older age groups and those who live rurally. There appears to be a specific need to promote safe handling techniques for dental care and grooming and canine desexing practices. Consequently, the present study offers a foundation for future research into the development of human behavior interventions in NZ, with the ultimate goal of boosting pet owners’ awareness of responsible pet owner practices within a One Welfare context and working towards all pets in NZ living a good life.

## Figures and Tables

**Table 1:** Percentage of respondents participating in the 2019 New Zealand Pet Survey indicating when their dog/s and cats are allowed inside.

Answer Choice	Dogs n (%)	Cats n (%)
Number of respondents	1449	1527
Whenever they choose (free access) during the day	962 (66.4%)	1307 (86%)
Whenever they choose (free access) at night	478 (33.0%)	850 (55%)
My pets are always confined indoors during the day	99 (6.8%)	92 (6%)
My pet is always confined indoors at night	573 (39.5%)	418 (27%)
Only when someone is at home	389 (26.8%)	87 (6%)
Other	167 (11.5%)	110 (7%)

**Table 2:** The percentage of positive responses to various dog care statements along with various factors and or variables that influence the likelihood (odds) of a positive response. The total number of responses was N = 1499.

Statement	n (%)	Associated Factor/s or Variable/s	Odds Ratio (95% CI, *p*-Value)
My dog is wormed according to product instructions	1373 (91.6%)	Gender: male/female	0.451 (0.245–0.831, 0.011)
My dog vaccinations are up to date	1358 (90.6%)	Number of children	0.814 (0.679–0.975, 0.026)
My dog is flea treated according to product instructions	1315 (87.7%)	Age range Town-living	0.878 (0.774–0.995, 0.042) 1.752 (1.209–2.539, 0.003)
My dog is desexed	1301 (86.8%)	Ethnicity Rural upbringing Town-living Number of adults Number of children	1.919 (1.310–2.810, 0.001) 0.683 (0.474–0.983, 0.040) 1.807 (1.259–2.595, 0.001) 0.783 (0.650–0.943, 0.010) 0.844 (0.719–0.991, 0.038)
My dog’s nails are clipped when needed	1210 (80.7%)	No associations	
My dog is groomed (brushed or clipped) regularly	748 (49.9%)	Town-living Number of children	1.310 (1.011–1.698, 0.041) 0.785 (0.691–0.892, <0.001)
My dog’s teeth are cleaned by the veterinarian when needed	634 (42.3%)	Ethnicity Household income Qualification level Number of children	1.349 (1.028–1.768, 0.031) 1.170 (1.055–1.298, 0.040) 0.934 (0.885–0.986, 0.013) 0.839 (0.734–0.958, 0.009)
My dog’s teeth are cleaned by me when needed	474 (31.6%)	Age range Household income	0.893 (0.819–0.974, 0.011) 0.822 (0.796–0.977, 0.016)
My dog has caused me financial stress (e.g., vet bills)	341 (22.8%)	Household income	0.858 (0.767–0.959, 0.017)

**Table 3:** The percentage of positive responses to various cat care statements along with various factors and or variables that influence the likelihood (odds) of a positive response. The total number of responses was N = 1536).

Statement	n (%)	Associated Factor/s and or Variable/s	Odds Ratio (95% CI, *p*-Value)
My cat is desexed	1490 (97.0%)	Qualification level Number of children	1.204 (1.034–1.401, 0.016) 0.681 (0.522–0.887, 0.004)
My cat is flea treated according to product instructions	1389 (90.4%)	Age range Town/city-dwelling	0.856 (0.739–0.991, 0.038) 1.934 (1.250–2.992, 0.003)
My cat is wormed according to product instructions	1388 (90.4%)	Gender Number of children	0.368 (0.189–0.720, 0.003) 0.757 (0.633–0.906, 0.002)
My cat vaccinations are up to date	1172 (76.3%)	Age range Rural upbringing	0.886 (0.806–0.974, 0.012) 0.738 (0.546–0.999, 0.049)
My cat’s teeth are cleaned by the veterinarian when needed	677 (44.1%)	Household income Qualification level Number of children Rural upbringing Town/city dwelling	1.265 (1.139–1.404, <0.001) 1.058 (1.001–1.117, 0.045) 0.648 (0.559–0.751, <0.001) 0.708 (0.536–0.936, 0.015) 1.411 (1.059–1.881, 0.019)
My cat’s nails are clipped when needed	546 (35.5%)	Household income Number of children Town/city-dwelling	1.125 (1.019–1.243, 0.020) 0.818 (0.712–0.941, 0.005) 1.369 (1.024–1.831, 0.034)
My cat is groomed (brushed or clipped) regularly	512 (33.3%)	Number of children	0.713 (0.611–0.833, <0.001)
My cat is groomed (brushed or clipped) occasionally	420 (27.3%)	Age range	0.862 (0.786–0.946, 0.002)
My cat has caused me financial stress (e.g., vet bills)	282 (18.4%)	Gender: male/female Age range Household income Number of adults Number of children	0.318 (0.113–0.892, 0.030) 0.855 (0.767–0.953, 0.005) 0.846 (0.751–0.954, 0.006) 0.818 (0.688–0.973, 0.023) 0.803 (0.669–0.965, 0.019)
My cat is a pedigree	144 (9.4%)	Qualification level	1.120 (1.025–1.225, 0.012)
My cat’s teeth are cleaned by me when needed	137 (8.9%)	Age range Household income Rural upbringing	0.771 (0.664–0.895, 0.001) 0.755 (0.638–0.892, 0.001) 1.656 (1.066–2.572, 0.025)

## Data Availability

The data presented in this study are available on request from the corresponding author.

## Supplementary Materials

The following supporting information can be downloaded at: https://www.mdpi.com/article/10.3390/ani13040631/s1, Supplementary Figure S1. The description and context of the overall survey; Table S1: demographic question from the Furry whānau wellbeing: Working with local communities for positive pet welfare outcomes survey.; Table S2: Thematic analysis of comments about when their dog or dogs are allowed inside by 2019 NZ Pet Survey respondents; Table S3: Thematic analysis of comments provided about when their cat or cats are allowed inside by 2019 NZ Pet Survey respondent.
