# Supplementary material for: Pet Ownership in Aotearoa New Zealand: A National Survey of Cat and Dog Owner Practices
Source: Animals (Basel). 2023 Feb 11;13(4):631. doi: 10.3390/ani13040631 (PMC9951667; doi:10.3390/ani13040631)
Supplement: Supplementary file 1 [file animals-13-00631-s001.zip › animals-2128438-supplementary.pdf]

animals MDPI

Supplementary Table S1: Demographic question from the Furry whānau wellbeing: Working with local communities for positive pet welfare outcomes survey.

Question	Possible Responses
Are you?	Female, Male, Gender Diverse
To which ethnic group do you belong? Tick as many as apply:	NZ European, Other European, Māori, Samoan, Cook Island Māori, Tongan, Niuean, Chinese, Indian, Don't know, Other (please specify)
To which age range do you belong?	18-24 years, 25-34 years, 35-44 years, 45-54 years, 55-64 years, 65-74 years, 75-84 years, 85 years and over
In which region do you currently live?	Northland, Auckland, Bay of Plenty, Waikato, Taranaki, Gisborne, Hawke's Bay, Manawatu-Wanganui, Wellington, Nelson, Marlborough, Tasman, West Coast, Canterbury, Otago, Southland, Other (please specify)
In the last 12 months, what was your total household income?	Less than $14,000, $14,001-48,000, $48,001-70,000, $70,001-100,000, Over $100,000, Would rather not say
What is your highest completed qualification?	National Certificate level 1, National Certificate level 2, National Certificate level 3, National Certificate level 4, Trade certificate, Diploma or Certificate level 5, Advanced trade certificate, Diploma or Certificate level 6, Teachers Certificate or Diploma, Nursing Diploma, Bachelor Degree, Bachelor (Hons), Postgraduate Certificate/Diploma, Master's Degree, PhD, Other (please specify)
Including yourself, how many adults (over 18 years of age) live at your usual address?	0, 1, 2, 3, Other (please specify)
How many children under 18 years of age live at your usual address?	0, 1, 2, 3, 4, 5, Other (please specify)
Were you born in New Zealand?	Yes, No
Where were you brought up? Tick as many as apply:	In a town or city, On a farm or rurally, On a lifestyle block, Other (please specify)
Where do you live now?	In a town or city, On a farm or rurally, On a lifestyle block, Other (please specify)
Do you own a dog?	Yes, No
How many dogs do you own?	1, 2, 3, 4, Other (please specify)
Do you own a cat?	Yes, No
How many cats do you own?	1, 2, 3, 4, Other (please specify)

Supplementary Table S2. Thematic analysis of comments about when their dog or dogs are allowed inside by 2019 NZ Pet Survey respondents.

Category	Theme	Subtheme	Representative quote/s
During the day	When someone is home	Free access	"Whenever they choose during the day when someone is at home."
		When invited	"Allowed inside when invited and supervised."
		Restricted freedom	"He is not allowed to roam around inside free range. He tells me if he needs something but otherwise is happy to stay in his bed near me."
		Constant companion	"My dog goes to work (and everywhere) with me."
	While no one is home	Free access	"They have the run of the house."
		Restricted access	"Has access to laundry and outside when I'm at work and the whole house when I'm home."
		Confined in the house (or part of)	"Confined indoors during the day while I'm at work."
		Crated inside	"They are crated inside if we are away."
		Confined in garage	"In the garage when no one is home."
		No inside access	"Always in a kennel outside if nobody is at home"
	During bad weather or health		"Kept indoors in inclement weather or when she has not been well"
	Never inside	Farm/working dog	"Farm dogs are outside"
		Pets	"Dog outside all day in fenced property"
During the night	When someone is home	Free access	"Free access night and day"
		Confined	"locked inside with us at night"
		Sleep in a designated area	"My dog sleeps on my bed and the old dog has her own bed"
		Crated	"At night one sleeps with my child and the other 2 are put in their cage for the night"
	During weather extremes		"Mostly an outdoor dog but joins us inside for evenings, and allowed inside when the weather is either too hot or too cold. Sleeps inside sometimes during winter months"
	Never inside	Outside	"Sleep outside"
		Designated area outside	"Secure in outside kennel for night time"

Supplementary Table S3. Thematic analysis of comments provided about when their cat or cats are allowed inside by 2019 NZ Pet Survey respondent.

Theme	Subtheme	Representative quote/s
Human intervention needed for inside access	No cat flap	"I rent and don't have a cat door"<br>"Allowed inside during day if someone is there or a window is open"
	Outside if no one home	"Current house doesn't have a cat door so not inside unless we are home"<br>"No cat flap so out during the day"
	Indoors if no one home	"Indoors when I'm not home but free access when I am home during the day"<br>"We don't have a cat door. When we are home (nights and weekends) she can go in/out as she wishes. When we are not home she is inside."
	Either in or out when no one home	"If we are out (work etc.) the house gets locked up wherever they are (inside or out) they stay"<br>"As long as someone is home she comes in and out. Due to no cat door at my rental, wherever she is when we leave home is where she stays until someone comes back"
Free inside access	Cat door	"Cat door/do as they please"<br>"They have free access 24/7 with cat doors"
Restricted inside access	When no one is home	"They have limited access when no one is home where they have food, water and creature comforts"<br>"Restricted to the conservatory generally when no one is home and at night time unless he is injured or sick"<br>"Free access to garage all the time"
Restricted outside access	At night	"Inside at night but let them out when they ask"<br>"Free access during the day and locked in at night/when it gets dark"<br>"When we go to bed whoever is inside stays inside but it's their choice"
	During extreme weather, illness or fireworks	"In the winter she is kept in at night but during the summer she has free access to inside/outside"<br>"During a storm or if they are sick"<br>"Fireworks- inside"
Always inside	Safety	"In the interest of keeping my cats safe, they are kept as indoor cats. They have a catio and we take them outside at least once a day for supervised outside time"<br>"She lives in house because it is not safe for a cat going outside in my hometown"
	Protect wildlife	"Cats kept indoors at all times to protect birdlife"
	Have cat conservatory or catio	"My girls are indoor cats and have access to a cat conservatory"<br>"They have a catio"
Won't come inside		"She won't come inside"<br>"If it is summer and hot sometimes the cat won't come in overnight"

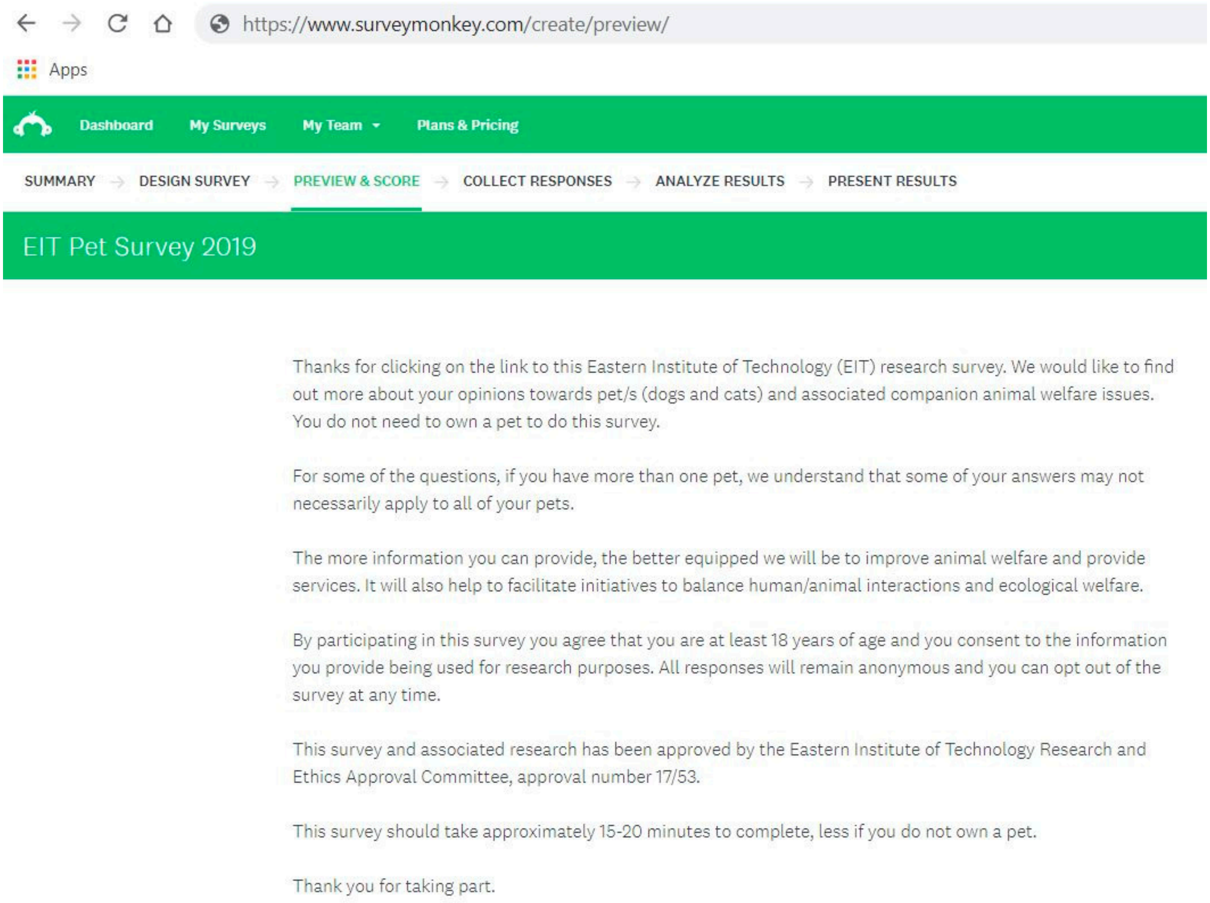

Supplementary Figure S1. The description and context of the overall survey.
